# Cloncurry buffel grass mitigated Cr(III) and Cr(VI) toxicity in tomato plant

**DOI:** 10.1038/s41598-022-25604-5

**Published:** 2022-12-05

**Authors:** Amna Shoaib, Saba Khurshid, Arshad Javaid

**Affiliations:** Department of Plant Pathology, Faculty of Agricultural Sciences, University of the Punjab, Quaid-e-Azam Campus, Lahore, Pakistan

**Keywords:** Plant sciences, Environmental sciences

## Abstract

Contamination of agricultural soil with chromium (Cr) ions has threatened global crop, human and ecosystem health. Its two oxidation states viz. Cr(III) and Cr(VI) are most stable and readily available to the plants. The study explored the impact of increasing exposure (up to 500 ppm) of Cr(III) and Cr(VI) on bio-physical traits of 15-day-old seedlings (in vitro) as well as 60-day-old tomato plant (in vivo), and highlighted the importance of buffel grass (*Cenchrus pennisetiformis*) in mitigating Cr levels in the tomato plants. In vitro*,* Petri plate bioassays with 13 different concentrations (20–500 ppm) of Cr(III) and Cr(VI) depicted the highly toxic effect of metal ions ≥ 200 ppm on all bio-physical traits of tomato seedlings. In vivo, soil spiked with Cr(III) and Cr(VI) (200, 300, and 400 mg/kg) was amended with 1% and 2% dry biomass of buffel grass. Phytotoxicity was higher in Cr(VI)-spiked soil compared with Cr(III)-spiked soil. Cr was mainly accumulated in tomato roots, and more Cr was translocated from roots to shoots from Cr(VI)-spiked soil than Cr(III)-spiked soil. Soil amendments with 2% weed biomass reduced metal toxicity in plants, particularly at 200 and 300 mg/kg of Cr. Protein profiles through SDS-PAGE revealed 12–50 kDa (mainly PR proteins) as an important region in tomato leaf, where many new bands were expressed under different treatments, particularly in the treatments provided with buffel grass. PCA-based biplot clearly separated Cr tolerance treatments from highly sensitive treatments. For the cultivation of tomato plants in Cr(III) and Cr(VI) contaminated soil (200 and 300 mg/kg), the biomass of Cloncurry buffel grass should be considered an effective and easily available phyto-management option.

## Introduction

Tomato (*Solanum lycopersicum* L.) belongs to the nightshade family Solanaceae and is a rich source of carotenoid (lycopene), vitamins (A, B, and C), phenolics, and energy reserves as mineral nutrients (P, N, K, Fe, Ca, Mg, etc.). Hence, its high nutritional profile includes it among widely grown crops worldwide especially in the USA, Mexico Spain, and Brazil, and in Asian countries like China, India, Iran, and Pakistan. Pakistan ranked 35th in tomato production around the globe, although, in 2019 recent production was 620.1 thousand tons from an area of 60.6 thousand hectares and it is expected to increase rapidly owing to the population’s increased demand^1^.

Soil is an amalgam of various contaminants including toxic heavy metals. The polluted soil adversely affects plant health and eventually leads to food insecurity and food safety issues^2^. Chromium (Cr) is a harmful heavy metal added to the soil, groundwater, and air mainly through industrial processes and then transferred to the plants, subsequently incorporated into the food chain at different and often multiple points, hence becoming the main risk factor for public health^3^. Therefore it is kept at 17th among the most hazardous substances^4^, and at level 1 among the carcinogenic elements^5^. Among its different oxidation states, Cr(VI) “hexavalent” and Cr(III) “trivalent” are the most common and stable states^6^, and a combination of both states is present in the soil. So far, both states differ concerning their bioavailability in soil, translocation, and toxicity within plants^7^. Cr(III) compounds are considered to be approximately 100 times less toxic than Cr(VI)^8^ and the latter state is frequently non-biodegradable, more soluble, more mobile, and may persist in the soil (particularly in sandy or low organic matter) for years^9^. The assimilation of Cr(III) by the plants occurs through passive diffusion^10^, while Cr(VI) is proposed to be absorbed through active diffusion via phosphate and sulfate transporters due to the structural similarity of Cr(VI) with these anions^11^.

It was also revealed that Cr(III) can be transformed to Cr(VI) inside the plant cell organs, and roots can accumulate is 100 times more Cr than other parts because the mobility of the Cr is low in the roots^12^. There is no evidence indicating the biological role of Cr in plant physiology^13^, hence both states can induce severe biochemical, ultrastructural, and molecular alterations in plants even at low concentrations of Cr by inducing oxidative stress^14–17^. Oxidative stress overwhelms the intrinsic anti-oxidant defenses, which contribute in the oxidation of lipid, protein, and nucleic acids leading to oxidative bursts, causing cellular damage, electrolyte leakage, and cell death^18^. Cr-induced cytotoxicity in the plants results in the disruption of signaling pathways and essential metabolic processes, and the plants manifest disorders associated with growth and yield in the plants^19–21^. The literature revealed that Cr-induced toxicity in *Brasicca* spp. *Triticum aestivum, Zea mays, Oryza sativa*, and *S. lycopersicum* etc. reduced/retarded the growth and biomass accumulation in these plants by increasing ROS accumulation, inhibiting cell division, the activity of vital enzymes, uptake and translocation of essential nutrients along with root injury, and leaf chlorosis^18–21^. Mangabeira et al.^22^ findings indicated that exposure of tomato plants to Cr(III) altered chloroplasts, and reduced numbers of grana and cristae, thus decreasing photosynthetic and respiratory activities with injurious consequences to plant health. Moreover, Cr(VI) toxicity causes bulbous outgrowths of nuclei and plastids, disruption of the vacuole, plastids, and Golgi bodies along with the aggregations of endoplasmic reticulum, and formation of lipid droplets in the cytoplasm^23^.

Therefore, there is a need to explore an effective alleviation of Cr toxicity in the plants, and the weeds belonging to *Cenchrus* species can be used for heavy metals stress mitigation^24–27^. The native *Cenchrus* species of the family Poaceae, respond quickly to rainfall events, produce more biomass than many native perennial grass species, and its high seed yields, hold the potential to grow in the industrial zone and mined land^28^. Among its different species, *Cenchrus pennisetiformis* commonly known as Cloncurry, white or slender buffel grass, is a fodder grass that is extremely drought tolerant, salt tolerant, and found as a dominantly growing weed worldwide. It contains proline, carbon isotopes, and malondialdehyde that make it a highly valuable herbicidal as well as heavy metals stress mitigator^29^. Considerable accumulation of different heavy metals has been documented in *Cenchrus* sp. growing in industrial contaminated zones due to the presence of sterols in its root^30–32^. Therefore, the present study was conducted to assess in vitro and in vivo toxicity caused by Cr(III) as well Cr(VI) on tomato plants and soil application with dry biomass of Cloncurry buffel grass (CPB) for Cr toxicity alleviation in the tomato plants.

## Results

### In vitro assays

**Figure 1:**
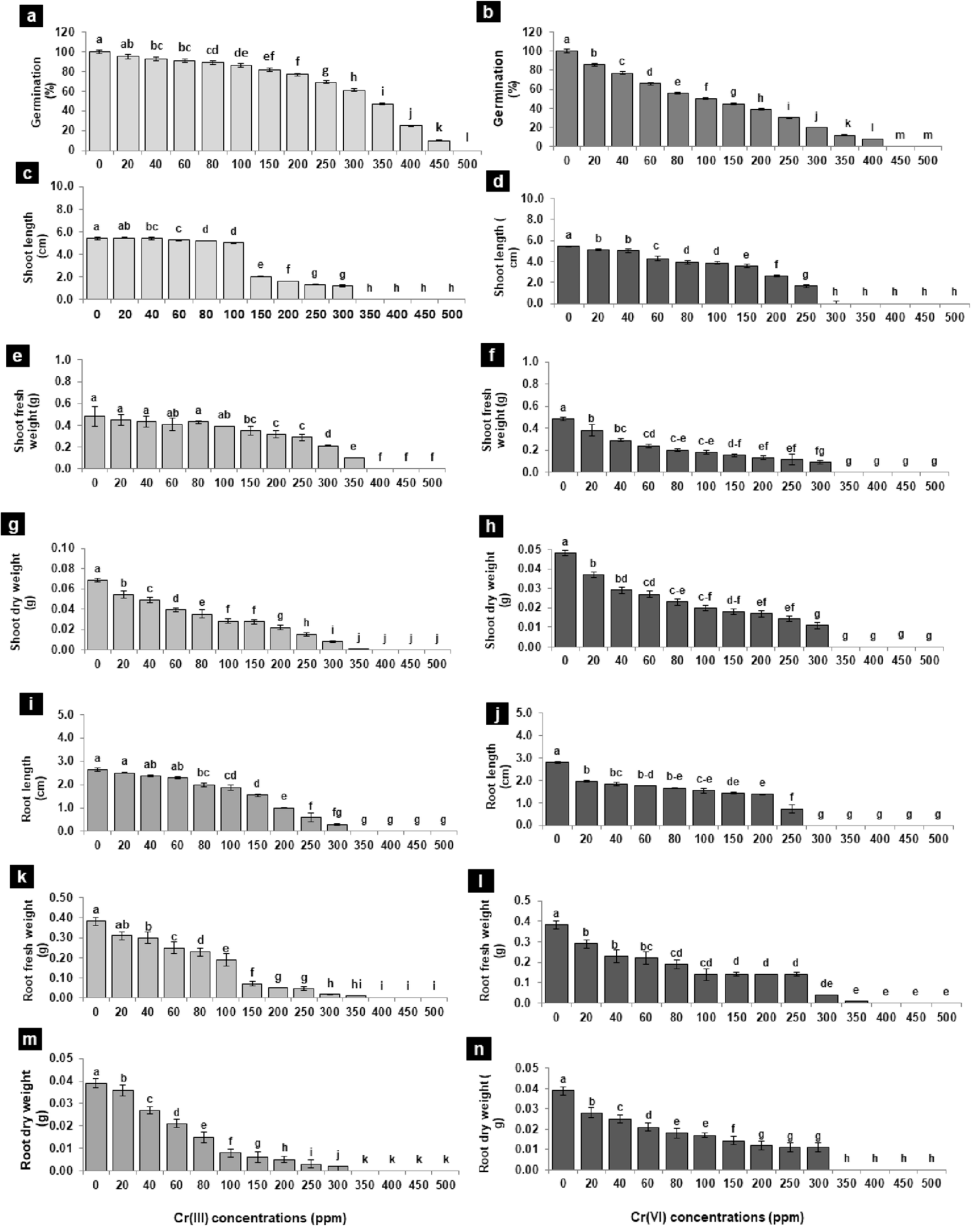
(**a**–**n**) Germination, seedling growth, and biomass of tomato due to the effect of 13 different concentrations of Cr(III) and Cr(VI) in Petri plate bioassays. Vertical bars show standard errors of the means of three replicates. Values with different letters at their top show a significant difference (p ≤ 0.05) as determined by the LSD test.

**Figure 2:**
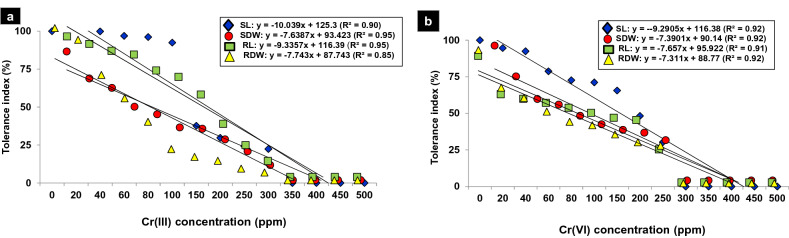
(**a** and **b**) Regression analysis for the relationship between tolerance indices (%) of tomato against 13 different concentrations of Cr(III) and Cr(VI) for 15-day-old tomato seedling in Petri plate bioassays.

**Figure 3:**
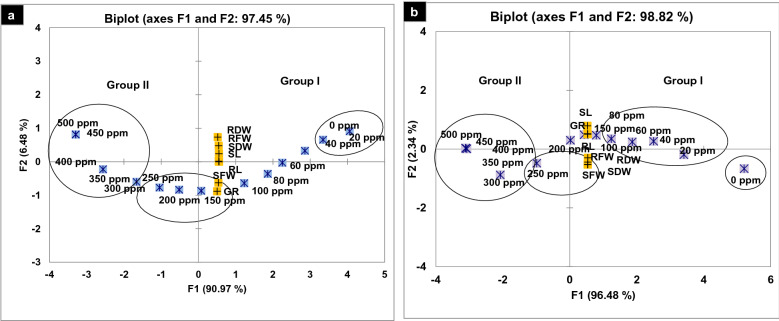
(**a** and **b**) Principal component analysis of germination, growth, and biomass due to the effect of 13 different concentrations of Cr(III) and Cr(VI) in Petri plate bioassays. *GR* Germination, *SL* shoot length, *RL* root length, *SFW* shoot fresh weight, *SDW* shoot dry weight, *RFW* root fresh weight, *RDW* root dry weight.

The impact of thirteen concentrations (20–500 ppm) of Cr(III) and Cr(VI) was adverse on the germination, seedling length, and dry biomass of 15-day-old tomato seedlings. The growth attributes were more sensitive toward different concentrations of Cr(VI) than to Cr(III). Therefore, germination was significantly decreased by 10–95% and 15–97% over a concentration range of 40–400 ppm of Cr(III) and 20–400 ppm of Cr(VI), respectively, while germination was completely halted beyond 400 ppm concentration. The seedling length and dry biomass decreased significantly by 10–90%, over a concentration range of 40–350 ppm of Cr(III) and 20–300 ppm of Cr(VI) and as compared to control (Fig. 1a–n). A negative linear relationship was found between the tolerance indices of the investigated attributes and the increasing metal concentrations with significantly greater R^2^ values (Fig. 2a,b). Likewise, the PCA-based biplot also distributed treatments into two major groups, with highly sensitive treatments being negatively correlated (p < 0.05) with all analyzed growth attributes present on the left side (200–500 ppm) of the biplot (Fig. 3a,b).

### In vivo assays

#### Growth assays

**Figure 4:**
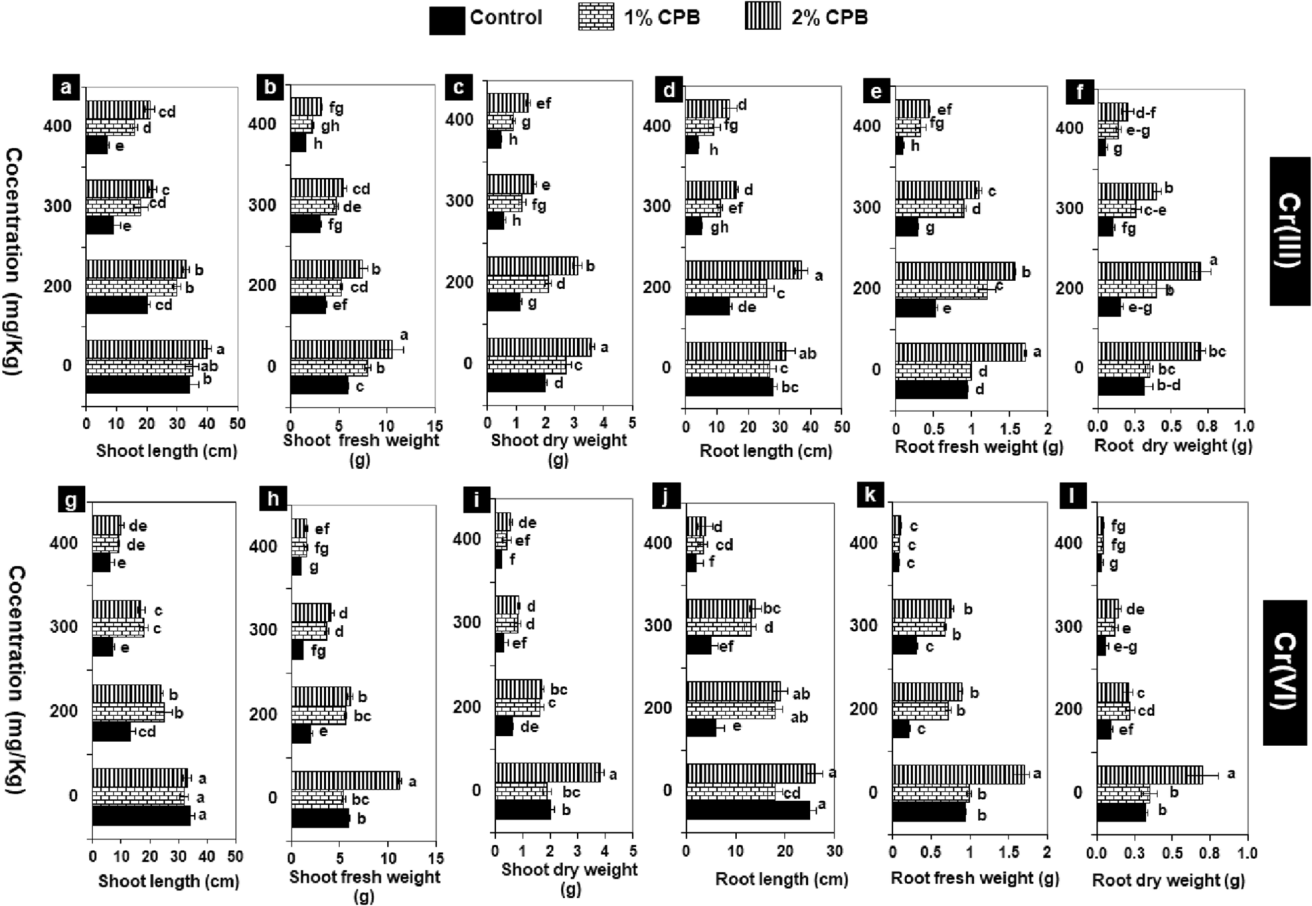
(**a**–**l**) Effect of soil amendment with plant biomass of Cloncurry buffel grass (CPB) on growth attributes of 60-day old tomato plant under Cr(III) and Cr(VI). Vertical bars show standard errors of the means of three replicates. Values with different letters at their top show a significant difference (p ≤ 0.05) as determined by the LSD test.

The plants in the control treatments exhibited significantly greater shoot/root length (34/28 cm), fresh biomass (5.90/0.94 g), and dry biomass (2.01/0.32 g), while, Cr(III)-spiked soil-induced toxicity in tomato plants by significantly decreasing shoot and root attributes by 40–80% and 50–90%, respectively with increasing Cr(III) concentrations (200, 300, and 400 mg/kg) (Fig. 4a–f). There was a more drastic reduction of 70–90% and 80–90% in the growth traits of shoot and root, respectively with elevating Cr(VI) concentration (200–400 mg/kg) in soil (Fig. 4g–l).

Soil mixing with CPB more profoundly affected the plant health with its 2% dose than 1% dose either under normal or Cr(III)-spiked soil. Though in the normal treatments, the plant length was affected insignificantly with either dose of CPB, biomass raised significantly up to 2 to 3-folds with 2% CPB as compared to corresponding control treatments (without metal). Mixing of 1% CPB in Cr(III)-spiked soil at 200, 300 and 400 mg/kg significantly improved all growth traits (length, fresh and dry biomass) of the shoot (1.5–2 folds) and root (2–3 folds) as compared to respective control [Cr(III) only]. Nevertheless, 2% CPB more remarkably enhanced shoot and root growth attributes by 2–3 folds and 3–4 folds, respectively with respect to the corresponding control of Cr(III) (Fig. 4a–f). Amendment of Cr(VI) contaminated soil with CPB was found effective for improving growth and biomass of tomato plants only at 200 and 300 mg/kg, while it failed to alleviate Cr(VI) stress significantly at 400 mg/kg. Hence, both lower dose (1%) and higher one (2%) of CPB exhibited statically similar improvement of 2–3 folds in the shoot as well as root growth attributes over corresponding control (Fig. 4g–l).

### SDS-PAGE

**Figure 5:**
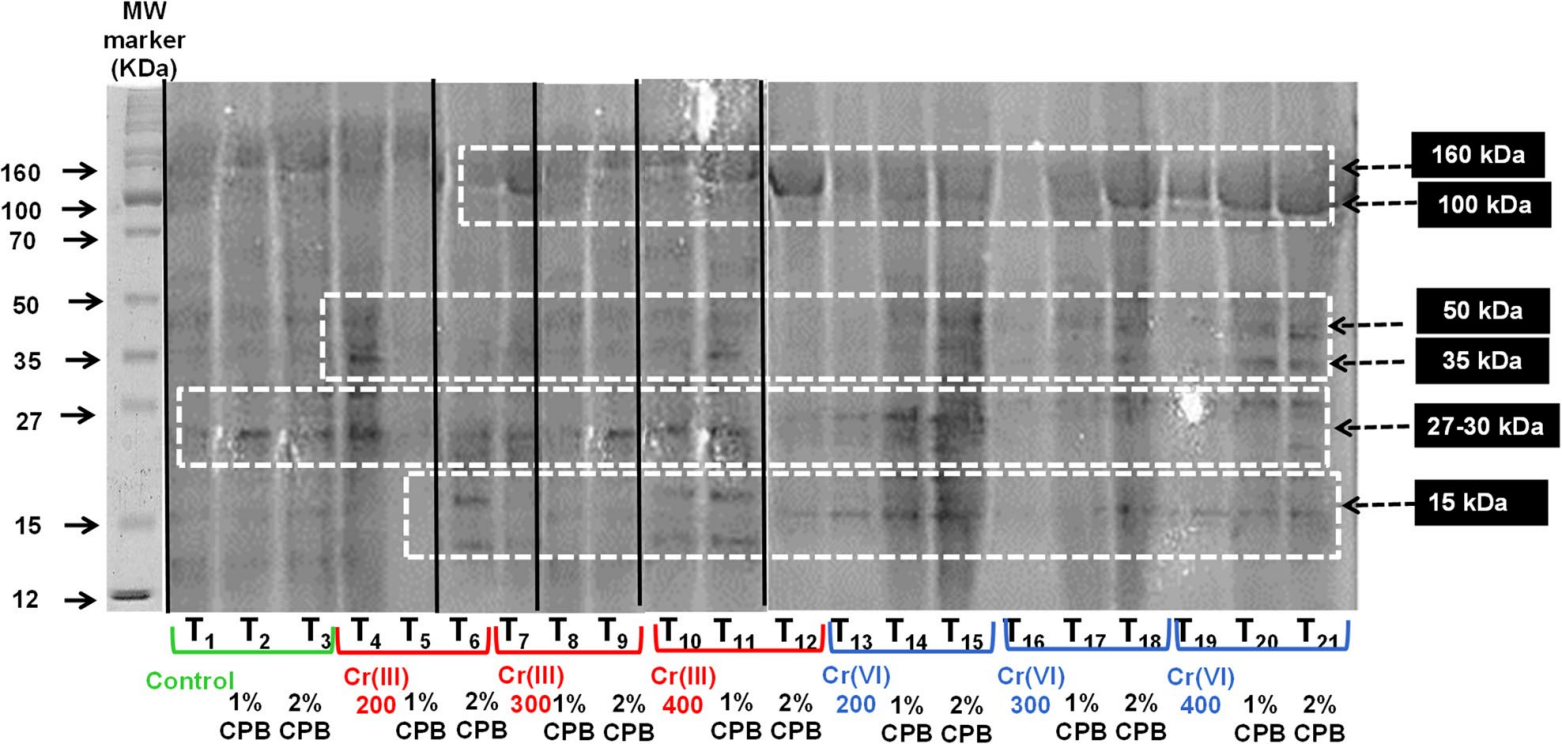
SDS-PAGE profile of tomato leaf due to the effect of soil amendment with plant biomass of Cloncurry buffel grass (CPB) under Cr(III) and Cr(VI) stress. The lanes report the MW marker, Molecular Weight protein ladder. T_1_: (Without Cr or CPB); T_2_: 1% CPB ; T_3_: 2% CPB mg/kg; T_4_: Cr(III) 200 mg/kg; T_5_:1% CPB + Cr(III) 200 mg/kg; T_6_: 2% CPB + Cr(III) 200 mg/kg; T_7_: Cr(III) 300 mg/kg; T_8_: 1% CPB + Cr(III) 300 mg/kg; T_9_: 2% CPB + Cr(III) 300 mg/kg; T_10_: Cr(III) 400 mg/kg; T_11_: 1% CPB + Cr(III) 400 mg/kg; T_12_: 2% CPB + Cr(III) 400 mg/kg; T_13_: Cr(VI) 200 mg/kg; T_14_: 1% CPB + Cr(VI) 200 mg/kg; T_15_: 2% CPB + Cr(VI) 200 mg/kg; T_16_: Cr(VI) 300 mg/kg; T_17_: 1% CPB + Cr(VI)300 mg/kg; T_18_: 2%CPB + Cr(VI) 200 mg/kg; T_19_: Cr(VI) 400 mg/kg; T_20_: 1% CPB + Cr(VI) 400 mg/kg; T_21_: 1% CPB + Cr(VI) 400 mg/kg. Full-length gel original image is available as supplementary data files in Fig. S1.

A considerable modification was also observed in the electrophoretic potential of tomato leaves in the treatments provided with Cr(III) and Cr(VI) stress as compared with the control. Many bands disappeared, while new bands were visible. In addition, there were significant polymorphisms in the mobility and number of low and high-molecular-weight proteins. All treatments exhibited protein bands at 100 and 160 kDa (*), although these bands were lightly stained in control, while these bands were intense and darkly stained in the treatments having metal stress or amended with CPB. Moreover, in comparison to the control, the protein bands at 70 kDa were weaker, while at 50–35 kDa appeared with a greater staining intensity in all treatments. Due to the Cr stress, some other polypeptides at 15–20 kDa molecular weight were observed with higher intensity. Several new bands at 35–50 kDa and 27–30 kDa in Cr-stressed plants provided with CPB showing stress alleviation (Fig. 5).

### Metal uptake by tomato plant

**Figure 6:**
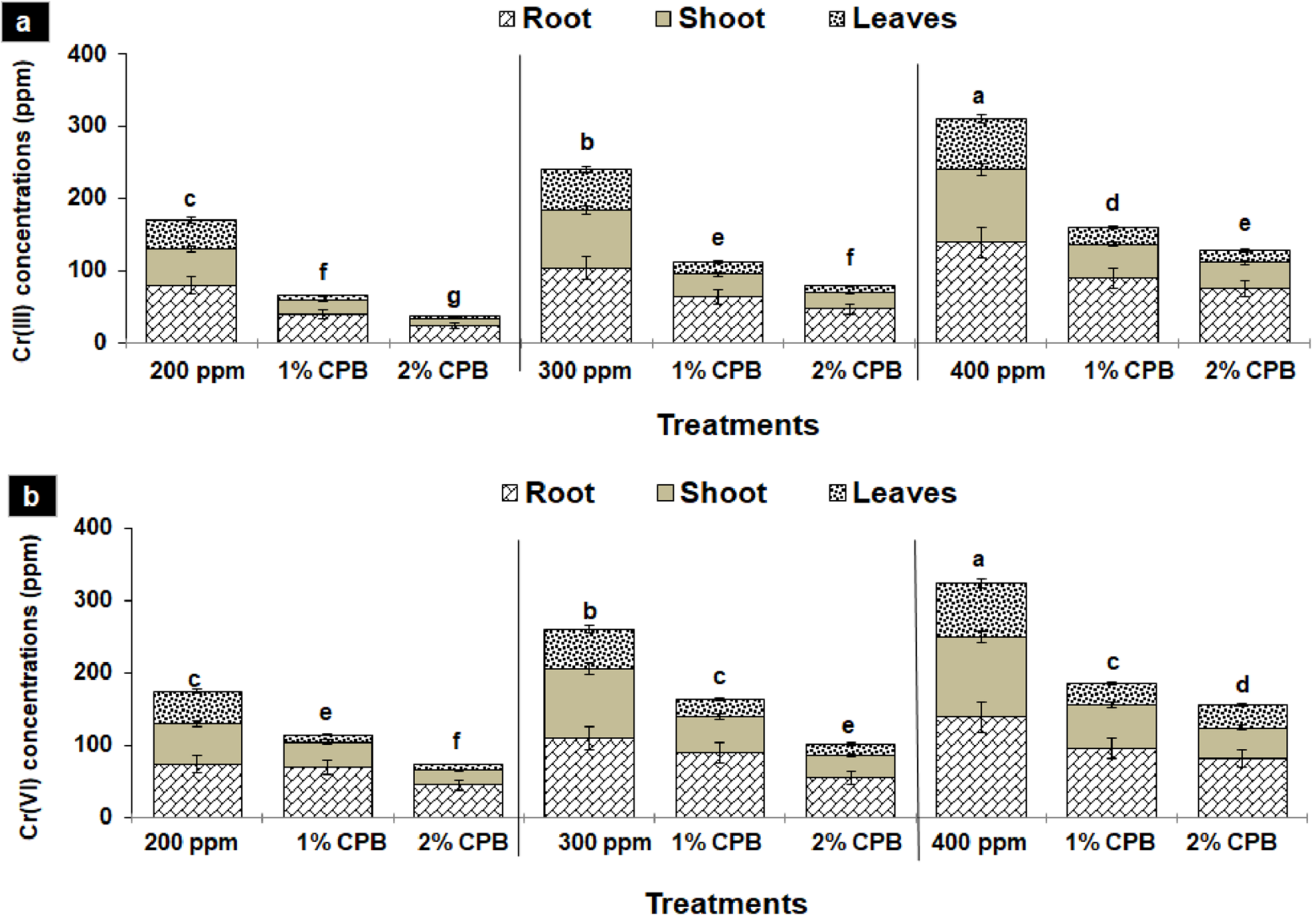
(**a** and **b**) Metal uptake by different parts of tomato plant due to soil amendment with plant biomass of Cloncurry buffel grass (CPB) under Cr(III) (a) and Cr(VI) (b) stress. Vertical bars show standard errors of the means of three replicates. Values with different letters at their top show a significant difference (p ≤ 0.05) as determined by the LSD test.

Generally, soil application of CPB remarkably lowered the metal ions uptake by tomato plants in a dose-dependent manner. The higher dose with 2% of the soil amendments proved to be more effective in lowering metal accumulation by different plant parts. The plant roots showed more potential to accumulate metal ions followed by the stem and then the leaves. Hence, a total Cr concentration of 170, 240, and 310 ppm were reduced significantly to 65, 112, and 161 ppm with the incorporation of 1% soil amendment in 200, 300, and 400 mg/kg Cr(III)-spiked soil, respectively. Nevertheless, the Cr accumulation by tomato plants further reduced significantly with 2% CPB, which resulted in the total accumulation of 36, 79, and 131 ppm in 200, 300, and 400 mg/kg Cr(III)-spiked soil, respectively (Fig. 6a).

Tomato plants showed more capacity to uptake Cr in the presence of Cr(VI) as compared to Cr(III), though CPB reduced metal uptake by tomato plants, uptake was still greater when the same was applied in Cr(III)-spiked soil. The tomato plants accumulated a total of 175, 261, and 325 ppm of total Cr at 200, 300, and 400 mg/kg of Cr(VI) in the absence of soil amendment, while accumulation reduced significantly to 115, 165, and 186 ppm with 1% CPB, and 74, 102 and 156 ppm with 2% CPB at the said Cr(VI) concentrations, respectively (Fig. 6b).

### Bioconcentration factor (BF) and translocation factor (TF)

The BF of 68/129, 163/632, and 285/310 indicated the greater efficacy of the tomato plant to accumulate Cr in its tissues in the presence of Cr(III)/Cr(VI) at 200, 300, and 400 mg/kg, respectively. However, the BF of 5–85 for Cr(III) and 22–212 for Cr(VI) was significantly less across all treatments amended with biomass of weed. Likewise, the TF values of all treatments were found to be less than 1 as compared to the treatments without soil amendments, which revealed more accumulation of metal ions in roots and slow transportation from the roots to shoots in the presence of CPB (Table 1).

**Table 1:** Translocation factor and bioconcentration factor of tomato leaf due to the effect of soil amendment with plant biomass of Cloncurry buffel grass (CPB) under Cr(III) and Cr(VI) stress.

Chromium	Concentration (mg/kg)	CPB	Translocation factor	Bioconcentration factor
Cr(III)	200		1.12b	64.86 g
		1% CPB	0.67ef	14.44j
		2% CPB	0.54 g	5.46 k
	300		1.31a	163.40d
		1% CPB	0.75e	41.38 h
		2% CPB	0.68ef	21.49i
	400		1.21ab	285.01c
		1% CPB	0.79d	83.52f.
		2% CPB	0.71ef	44.56 h
Cr(VI)	200		1.36a	111.12e
		1% CPB	0.64f.	36.11 h
		2% CPB	0.64f.	22.21i
	300		1.37a	310.99b
		1% CPB	0.83d	93.36f.
		2% CPB	0.85d	53.72 g
	400		1.32a	576.83a
		1% CPB	0.94c	212.01d
		2% CPB	0.90c	135.44e

Values with different letters (n = 3) at their top show a significant difference (p ≤ 0.05) as determined by the LSD test.

### PCA-biplot

**Figure 7:**
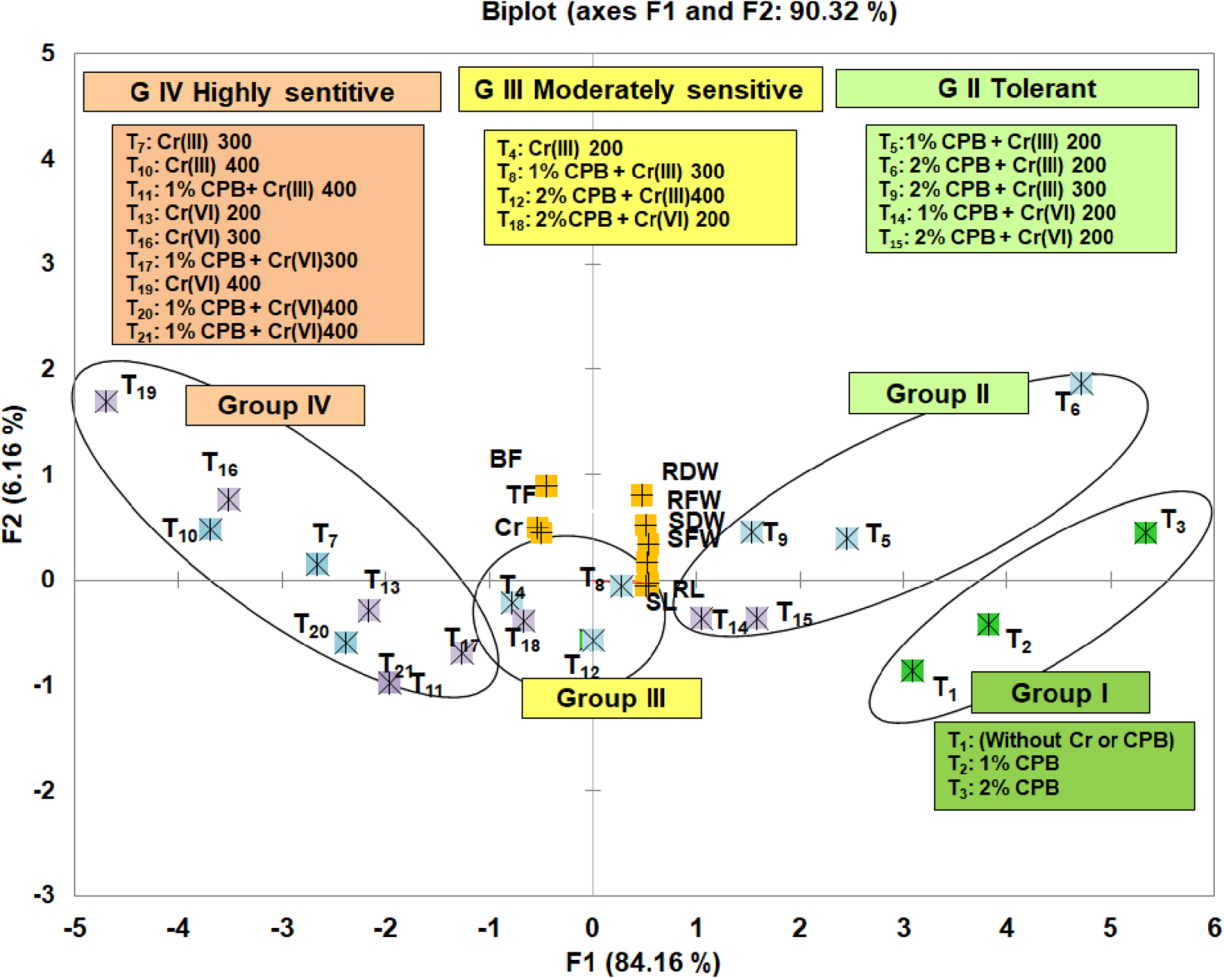
Principal component analysis of biophysical and metal accumulation attributes in 60-day-old tomato plants due to the effect of plant biomass of Cloncurry buffel grass (CPB), Cr(III), and Cr(VI). *SL* shoot length, *RL* root length, *SFW* shoot fresh weight, *SDW* shoot dry weight, *RFW* root fresh weight, *RDW* root dry weight, *TF* translocation factor, *BF* bioconcentration factor, *Cr* chromium accumulation.

A PCA was performed to identify the association of variables with each other and their effect on the treatments. PC1 and PC2 accounted explained 89.30% of the variability in the data among all traits tested in this study (Fig. 7). PC1 mainly correlated all growth attributes, and PC2 described the correlation of metal accumulation, translocation factor, and bioconcentration factor, but the vectors of PC1 and PC2 pointed in the opposite directions, demonstrating a negative correlation. Four groups are made on the basis of the response to treatments. Group I, consisted of control (T_1_) along with treatments who received 1% CPB (T_2_) and 2% CPB (T_3_) with significantly greater growth attributes. Highly sensitive treatments towards Cr(III) (T_7_ and T_10_) and Cr(VI) (T_13_, T_16_ and T_19_) along with 1% CPB + Cr (III and VI) at higher concentrations were placed in group IV on the left side of the biplot, while sensitivity increased as treatments placed away from the origin. In other treatments, weed biomass posed against Cr menace and placed the 5 tolerant treatments [T_5_, T_6_, T_9_, T_14_ and T_15_] on the right side of the biplot (group II), while 4 moderately tolerant treatments [T_4_, T_8_, T_12_ and T_18_] in the middle of the biplot (group III) as compared to control (group I) (Fig. 7).

## Discussion

The toxic level of heavy metal Cr in agricultural soil has become a worldwide public health concern, and, there is still a need to apply easy, eco-friendly, green, sustainable, and economic strategies to address this issue. Buffel grass (*C. pennisetiformis*) exhibits wide climatic tolerance, and utilizing its dry biomass in addressing metal toxic levels in soil may be helpful mitigating in soil Cr toxicity^32^. Preliminary, in vitro assays with 13 concentrations (20, 40, 60, 80, 100, 150, 200, 250, 300, 350, 400, 450, and 500 ppm) of each Cr(III) and Cr(VI) exhibited a significant reduction in germination and growth in 15-day-old tomato seedlings, while the toxic effect increased with increasing Cr concentrations (≥ 200 ppm). Moreover, the seeds were unable to germinate beyond 400 ppm of the Cr with up to 90% reduction in the seedlings’ length and biomass. Regression analysis and PCA-biplot also confirmed the inhibitory effect of higher concentrations ≥ 200 ppm of Cr(III) and Cr(VI). Further, in vivo experimentation with 200, 300, and 400 mg/kg of Cr(III) and Cr(VI) further revealed a significant decline of 40–90% and 70–90%, respectively in growth traits (length, fresh and dry biomass) of the 60-day-old tomato plant as compared to control.

In plants, seed germination is the first exchange interface with the surrounding, when germination is under Cr stress, the reduction in seed viability has been linked with damage to nucleic acid and membranes through over-accumulation of ROS, which may decrease oxygen uptake and immobilization of reserve food materials for growth^33^. The interplay between ROS and major signaling components (calcium-signaling steers, mitogen-activated kinases, and hormonal signaling), activates remote signaling pathways to translate metal-induced oxidative stress into highly specific cellular signals in the plants. ROS by inducing macromolecule deterioration, membrane dismantling, and leaking of ions leads to lethal effects in plants^34^. Oxygen depletion, along with disruption in the transport of water and mineral (e.g. Ca, K and Mg), and abnormal cell division in roots have been documented^35^, which in turn by decreasing total biomass, eventually reduced plant yield^36^. Additionally, damage to root tip cells has accounted for Cr accumulation in root cells, and ultrastructural damage in leaf mesophyll cells has been linked with a decline in shoot development^37^. Growth seizing, inhibition in biomass, and changes in physicochemical attributes of the plants under the higher concentrations of Cr have been noticed formerly in tomato, mustard, chickpea, mungbean, and brinjal plants^36–38^.

Cr toxicity in plant systems and its physiological modulation mainly depend on the quantity of Cr uptake by a plant, its mobilization, and subsequent accumulation in various tissues^39^. It was found that the uptake of active-redox Cr(VI) by the tomato plants was greater than the uptake of Cr(III), which might be attributed to strong oxidizing potential, more mobility, and availability of Cr(VI) to the plant^25^. Besides, Cr(VI) is easily transported to other parts of the plant as it takes phosphate and sulfate pathways, rather Cr(III) is generally transported through an inactive pathway^37^. Furthermore, total Cr accumulation in all plant parts was increased with an increasing Cr concentration, which also raised the translocation factor [Cr(III):1.12–1.3 and Cr(VI): 1.32–1.37] and bioconcentration factor [Cr(III): 70–290 and Cr(VI): 100–580]. Moreover, the root accumulated greater Cr than the stem and leaf, it was also revealed by TF and BCF. Nonetheless, TF values greater than 1 indicated higher metal translocation from the root to above-ground parts of the plant, while higher BCF showed metal concentration in plant tissues relative to the growth medium^40^. The results are in accordance with older reports, where higher Cr accumulation in the root has been associated with metal immobilization in the root cells rendering it less toxic for aerial parts^41^.

Soil mixing with CPB markedly improved the plant growth and biomass (1–4 folds) by decreasing Cr uptake in the tomato plants in a dose-dependent manner, as the 2% CPB was more effective than the 1% CPB. Hence, a significant reduction in the total Cr concentration in the different parts of the tomato plant also caused low values of TF and BCF in the presence of CPB. As Cr accumulation was mostly confined to the root, while roots were in contact with biomass of *C. pennisetiformis*. The robust nature of halophytic buffel grass makes it a suitable candidate for binding Cr ions as reported in the previous study, where ethyl-acetate sub-fraction of the methanolic shoot extracts of *C. pennisetiformis* indicated the occurrence of ethanone 1-[2,4,5 triethoxyphenyl]; eucalyptol; hexadecanoic acid, ethyl-ester; 2,3-dihydro 1-benzofuran; 1-propanol-2-2-hydroxypropxy; 1-eicosene and E-15-heptadecenal]^16^. These compounds may be responsible for metal chelation, complexation, electrostatic interaction, and cations exchange with other mechanisms. Moreover, these compounds in *C. pennisetiformis* can help in directly scavenging ROS in plants under stress^42^. Ghoneim et al.^43^ also explored the accumulation of high concentrations of many heavy metals including Cr by *Cenchrus ciliaris* due to the high content of phenolics and other antioxidants. Likewise, Nazir et al.^30^ also documented the accumulation of high concentrations of the heavy meal by the roots of *C. pennisetiformis* due to a higher affinity for metal ions. The occurrence of cycloergost, phytol, and β-tocopherol in the root extract of different *Cenchrus* spp. has been shown to exhibit metal scavenging action^31^. Therefore, a decrease in Cr accumulation increased tomato plant tolerance by boosting its growth and biomass by soil application of CPB^25^.

Posttranslational variations analyzed through SDS-PAGE exhibited biochemical and structural adjustments in tomato plants after metal exposure with or without CPB, probably due to the synthesis of stress-proteins as well as heat shock proteins imperative phytochelatin biosynthesis^44^. Under the Cr stress, protein bands with higher intensity were observed at 160 kDa (cytochrome c oxidase, complex IV), while their function seems to restore after soil amendment with CPB. Another band belonging to glucoproteins (70 kDa) exhibited less intensity across all treatments as compared to the control. Interestingly, 12–50 kDa (apoplastic proteases and defense responses-PR proteins) appears as an important region in the tomato leaf, because the expression of many bands intensified in this region after Cr exposure, while the same bands exhibited normal expression along with the formation of some new bands after soil mixing with CPB. A key role of PR proteins [chitinases (PR-3 family), β-1,3-glucanases (PR-2 family), and thaumatin-like protein (PR-5 family)] has been documented in plant adaptation to stressful environments^45,46^. Likewise, changes in the region of 17–15 KDa (PR-10) were in harmony with the previous findings as the protein of this region 16 kDa could react with different metal ions after metal exposure^43^.

All three PCA explained ≥ 90% of the data variability^47^. Factor-loading matrix extracted from biplot analysis of all PCA derived from in vitro and in vivo studies indicated, a negative correlation of the studied growth attributes of tomato plants with increasing concentration of Cr^48^. Moreover, in vivo metal accumulation, translocation factor, and bioconcentration factor are positively correlated with the treatments in the highly sensitive groups. Besides, all treatments in group II are located near the control group, which presented the significance of Cloncurry grass as a soil amendment in alleviating Cr stress^25^. Therefore, mixing of CBP biomass or Cloncurry grass could be utilized to alleviate the Cr toxicity in tomato plants growing under the toxic concentration of Cr particularly at 200 and 300 mg/kg.

## Conclusions

In vitro and in vivo bioassays showed that tomato germination and seedling growth were highly sensitive to 200–400 mg/kg of Cr(III) and Cr(VI) ions concentrations. However, soil amendments with 2% biomass of *C. pennisetiformis* showed more remarkable results in decreasing metal toxicity at 200 and 300 mg/kg of metal ions. Changes observed through protein profiles were well-linked with growth assays. Soil amendment with 2% plant biomass of Cloncurry could be used to reduce Cr toxicity in soil within the concentration range of 200–300 mg/kg of metal ions by improving tomato plant health. This study would be a milestone towards a solution to intricate environmental problems caused by carcinogenic heavy metal viz., Cr using the Cloncurry buffel grass.

## Materials and methods

### Laboratory bioassays

The seeds of tomato variety LA-2662 were supplied by Vegetable Research Institute, Ayub Agriculture Research Institute (Faisalabad, Pakistan). Experimental research on plants is in compliance with relevant institutional, national, and international guidelines and legislation. Thirteen concentrations viz. 20, 40, 60, 80, 100, 150, 200, 250, 300, 350, 400, 450, and 500 ppm of each Cr(III) and Cr(VI) were prepared from the stock solution (1000 ppm) was prepared using chromium nitrate [Cr(NO_3_)_3_ 9H_2_O] and potassium dichromate (K_2_Cr_2_O_7_), respectively. Pre-sterilized Petri plates (9-cm diameter) were lined with a single layer of sterilized filter paper and 25 healthy, surface sterilized seeds of tomato var. LA-2662 was placed on each plate. Seeds were moistened with 3 mL of each of the 13 concentrations of Cr(III) and Cr(VI) separately. Control Petri plates were prepared by pouring 3 mL of distilled water on the seeds. The quadruple set of 29 treatments was set in a growth chamber at 25 ± 2 °C with 10 h light period for 15 days. The percentage of germinated seeds, length, fresh, and dry weight of seedlings were recorded 15 days after germination. The dry weight of the seedlings was determined after oven drying at 70 °C till constant weight.

### Pot trials

A greenhouse experiment was conducted in earthen pots (20 cm diameter, 30 cm height) in the Experimental Station of the Faculty of Agricultural Sciences, University of the Punjab Lahore, Pakistan. Three concentrations viz. 200, 300, and 400 mg/kg of each Cr(III) and Cr(VI) were selected from the laboratory screening trials to conduct pot trials. The soil was spiked with 200, 300, and 400 mg/kg of Cr(III) and Cr(VI), and non-spiked soil was taken as control. For homogenization and drying, the soil was left for 15 days. Then metal-spiked potted soil was mixed with biomass of Cloncurry buffel grass (CPB). Whole plants of buffel grass were collected from Lahore, Pakistan, according to prescribed rules in The Pakistan Trade Control of Wild Fauna and Flora Act, 2012. It was identified by Prof. Dr. Arshad Javaid (Faculty of Agricultural Sciences, University of the Punjab, Lahore, Pakistan), assigned voucher no. GC. Bot. Herb. 825, and was deposited in the Dr. Sultan Ahmed Herbarium, Department of Botany, GC University, Lahore, Pakistan. The plants were washed, dried at 45 °C, powered, and thoroughly mixed @ 1% and 2% (w/w) in potting soil. Tomato variety LA-2662 seedlings with 4–5 leaves were transplanted (3 seedlings per pot). A completely randomized experiment with a triplicate set of 21 treatments (Table 2) was placed in a greenhouse (25 °C ± 3; 12 h photoperiod and 70% relative humidity) and moisture level was maintained to field capacity by irrigating with tap water whenever required.

**Table 2:** Treatments of the experiment for assessing the effect of plant biomass of Cloncurry buffel grass (CPB) in alleviating Cr toxicity in tomato plants.

Metal	Concentration	0	1% CPB	2% CPB
	0	T_1_	T_2_	T_3_
Cr(III) mg/kg	200	T_4_	T_5_	T_6_
	300	T_7_	T_8_	T_9_
	400	T_10_	T_11_	T_12_
Cr(VI) mg/kg	200	T_13_	T_14_	T_15_
	300	T_16_	T_17_	T_18_
	400	T_19_	T_20_	T_21_

All the 21 treatments were analyzed for changes in the electrophoretic profile of protein through SDS-PAGE, growth attributes and metal accumulation.

### Protein profiling by SDS-PAGE

Total protein was isolated from the leaf samples of the 4-week-old plant (300 mg)^49^. Protein samples were run on 10% SDS-PAGE gels (separating gel: 1.5 M Tris pH 8.8, 10% SDS (w/v), 30% (v/v) acrylamide, 10% (w/v) (NH_4_)_2_S_2_O_8_, 0.05% (v/v) TEMED; stacking gel: 1 M Tris pH 6.8, 10% (w/v) SDS, 30% acrylamide, 10% (NH_4_)_2_S_2_O_8_, 0.01% (v/v) TEMED). About 2 μL of this protein was mixed with 8 μL 1 × running buffer loading dye (60 mM Tris pH 6.8, 25% (v/v) glycerol, 5% (w/v) SDS, 1% (v/v) saturated bromophenol blue). After incubating for 30 min at room temperature, the protein was run in 1 × SDS running buffer (250 mM Tris, pH 8.3, 500 mM glycine, 1% (w/v) SDS) at 200 V with a protein size marker, until the dye was 1–2 mm from the end of the gel. The gel was stained in a Coomassie Blue stain solution (0.1% (w/v) Coomassie brilliant blue, 45% (v/v) methanol, and 10% (v/v) acetic acid) for 20–30 min and then washed with PAGE-destain (10% (v/v) acetic acid, 45% (v/v) ethanol) for several times to visualize protein. The gels were transilluminated by LED light (DaiHan WUV-L50, Korea) and images were captured with a digital camera (Canon 850D).

### Growth assays and metal analysis

After 60 days of sowing, length, fresh and dry biomass of plants in 21 treatments were recorded. The dried root, stem, and leaf samples of the plants were powdered, and digested separately using 2 mL 70% v/v nitric acid at 100 ºC for 2 h exposures^50^ and analyzed for total Cr concentration through Atomic absorption spectroscopy (Thermo scientific ICE 3000 SERIES). The translocation factor was calculated by the following equation:

$${\text{TF}} = {\text{Cshoot/Croot}}$$

where Cshoot and Croot are metals concentration in the shoot and root of the plant, respectively. TF > 1 represents that translocation of metals effectively was made to the shoot from root^51^. The bioconcentration factor (BCF) was calculated using the following formula^52^:

$${\text{BCF}}\,\left( {\text{\%}} \right) = {\text{ SMC}} \times {\text{SDW}} + {\text{RMC}} \times {\text{RDW}}\,\left( {\text{A}} \right) = {\text{ SMC}} \times {\text{RMC/A}}$$

 ∗ Trait: SMC: shoot metal concentration, SDW: shoot dry weight; RMC: root metal concentration, RDW: roots dry weight.

### Statistical analysis

Data of phenotypic attributes were analyzed through LSD test (p ≥ 0.05) was applied to identify significant differences using Statistics 8.1. Data of in *vitro assays* was compared by drawing trend lines best fit the data. Principal components analysis was performed to summarize the variability of the treatments and to determine the association among the measured traits.

### Ethical approval

All procedures in this experiment were carried out in accordance with relevant guidelines of the university field of the University of the Punjab, Lahore, Pakistan.

## Supplementary Information

Supplementary Information.

## Data Availability

The original unscaled image for Fig. 5 is available as a supplementary file (Fig. S1). All the raw data is provided in a supplementary file and the datasets used and/or analyzed during the current study are available (Table S1–S6) from the corresponding author on reasonable request.
