# Supplementary material for: Cloncurry buffel grass mitigated Cr(III) and Cr(VI) toxicity in tomato plant
Source: Sci Rep. 2022 Dec 5;12:20952. doi: 10.1038/s41598-022-25604-5 (PMC9723175; doi:10.1038/s41598-022-25604-5)
Supplement: Supplementary file 1 — Supplementary Information. [file 41598_2022_25604_MOESM1_ESM.docx]

**Figure S1.** Full-length gel of tomato leaf due to the effect of soil amendment with plant biomass of Cloncurry buffel grass (CPB) under Cr(III) and Cr(VI) stress. The lanes report the MW marker, Molecular Weight protein ladder (BenchMark). T_1_: (Without Cr or CPB); T_2_: 1% CPB ; T_3_: 2% CPB mg/Kg; T_4_: Cr(III) 200 mg/Kg; T_5_:1% CPB + Cr(III) 200 mg/Kg; T_6_: 2% CPB + Cr(III) 200 mg/Kg; T_7_: Cr(III) 300 mg/Kg; T_8_: 1% CPB + Cr(III) 300 mg/Kg; T_9_: 2% CPB + Cr(III) 300 mg/Kg; T_10_: Cr(III) 400 mg/Kg; T_11_: 1% CPB+ Cr(III) 400 mg/Kg; T_12_: 2% CPB + Cr(III) 400 mg/Kg; T_13_: Cr(VI) 200 mg/Kg; T_14_: 1% CPB + Cr(VI) 200 mg/Kg; T_15_: 2% CPB + Cr(VI) 200 mg/Kg; T_16_: Cr(VI) 300 mg/Kg; T_17_: 1% CPB + Cr(VI)300 mg/Kg; T_18_: 2%CPB + Cr(VI) 200 mg/Kg; T_19_: Cr(VI) 400 mg/Kg; T_20_: 1% CPB + Cr(VI) 400 mg/Kg; T_21_: 1% CPB + Cr(VI) 400 mg/Kg.

**Table S1:** Germination, seedling growth, and biomass of tomato due to the effect of 13 different concentrations of Cr(III) and Cr(II) in Petri plate bioassays.

| **Treatments** | **Concentration**  **(ppm)** | **Germination**  **(%)** | **Shoot length**  **(cm)** | **Shoot fresh weight**  **(g)** | **Shoot dry**  **weight**  **(g)** | **Root**  **length**  **(cm)** | **Root**  **fresh weight**  **(g)** | **Root**  **dry**  **weight**  **(g)** |
| --- | --- | --- | --- | --- | --- | --- | --- | --- |
| **T_1_** | **0** | 100 | 5.41 | 0.48 | 0.068 | 2.64 | 0.38 | 0.039 |
| **T_2_** | **20** | 95 | 5.46 | 0.45 | 0.054 | 2.5 | 0.31 | 0.036 |
| **T_3_** | **40** | 93 | 5.4 | 0.43 | 0.049 | 2.37 | 0.3 | 0.027 |
| **T_4_** | **60** | 91 | 5.24 | 0.41 | 0.039 | 2.3 | 0.25 | 0.021 |
| **T_5_** | **80** | 89 | 5.2 | 0.43 | 0.035 | 2 | 0.23 | 0.015 |
| **T_6_** | **100** | 86 | 5 | 0.39 | 0.028 | 1.88 | 0.19 | 0.008 |
| **T_7_** | **150** | 82 | 2.03 | 0.35 | 0.027 | 1.55 | 0.070 | 0.006 |
| **T_8_** | **200** | 77 | 1.61 | 0.32 | 0.021 | 1 | 0.05 | 0.005 |
| **T_9_** | **250** | 69 | 1.31 | 0.29 | 0.015 | 0.6 | 0.045 | 0.003 |
| **T_10_** | **300** | 61 | 1.21 | 0.21 | 0.008 | 0.3 | 0.017 | 0.002 |
| **T_11_** | **350** | 47 | 0.3 | 0.1 | 0.001 | 0.1 | 0.01 | 0 |
| **T_12_** | **400** | 0 | 0 | 0 | 0 | 0 | 0 | 0 |
| **T_13_** | **450** | 0 | 0 | 0 | 0 | 0 | 0 | 0 |
| **T_14_** | **500** | 0 | 0 | 0 | 0 | 0 | 0 | 0 |

Values are the mean of three replicates

**Table S2:** Germination, seedling growth, and biomass of tomato due to the effect of different concentrations of Cr(VI) in Petri plate bioassays.

| **Treatments** | **Concentration**  **(ppm)** | **Germination**  **(%)** | **Shoot length**  **(cm)** | **Shoot fresh weight**  **(g)** | **Shoot dry**  **weight**  **(g)** | **Root**  **length**  **(cm)** | **Root**  **fresh weight**  **(g)** | **Root**  **dry**  **weight**  **(g)** |
| --- | --- | --- | --- | --- | --- | --- | --- | --- |
| **T_1_** | **0** | 100 | 5.41 | 0.48 | 0.048 | 2.8 | 0.38 | 0.039 |
| **T_2_** | **20** | 86 | 5.12 | 0.38 | 0.037 | 1.95 | 0.29 | 0.028 |
| **T_3_** | **40** | 77 | 5 | 0.29 | 0.029 | 1.85 | 0.23 | 0.025 |
| **T_4_** | **60** | 66 | 4.26 | 0.24 | 0.027 | 1.76 | 0.22 | 0.021 |
| **T_5_** | **80** | 56 | 3.93 | 0.2 | 0.023 | 1.65 | 0.19 | 0.018 |
| **T_6_** | **100** | 50 | 3.85 | 0.18 | 0.02 | 1.54 | 0.14 | 0.017 |
| **T_7_** | **150** | 45 | 3.55 | 0.15 | 0.018 | 1.43 | 0.142 | 0.014 |
| **T_8_** | **200** | 39 | 2.61 | 0.13 | 0.017 | 1.38 | 0.142 | 0.012 |
| **T_9_** | **250** | 20 | 0.67 | 0.1 | 0.01 | 0.45 | 0.05 | 0.005 |
| **T_10_** | **300** | 0 | 0 | 0 | 0 | 0 | 0 | 0 |
| **T_11_** | **350** | 0 | 0 | 0 | 0 | 0 | 0 | 0 |
| **T_12_** | **400** | 0 | 0 | 0 | 0 | 0 | 0 | 0 |
| **T_13_** | **450** | 0 | 0 | 0 | 0 | 0 | 0 | 0 |
| **T_14_** | **500** | 0 | 0 | 0 | 0 | 0 | 0 | 0 |

Values are mean of three replicates

**Table S3:** Effect of soil amendment with plant biomass of Cloncurry buffel grass (CPB) on growth attributes of 60-day old tomato plant under Cr(III).

| **Treatments** | **Concentration** | **Shoot length**  **(cm)** | **Shoot fresh weight**  **(g)** | **Shoot dry**  **weight**  **(g)** | **Root**  **length**  **(cm)** | **Root**  **fresh weight**  **(g)** | **Root**  **dry**  **weight**  **(g)** |
| --- | --- | --- | --- | --- | --- | --- | --- |
| **T_1_** | **0** | 34.0 | 5.9 | 2.0 | 28.0 | 0.9 | 0.3 |
| **T_2_** | **200 (mg/Kg)** | 20.0 | 3.7 | 1.1 | 14.3 | 0.5 | 0.2 |
| **T_3_** | **300 (mg/Kg)** | 9.0 | 3.1 | 0.6 | 5.0 | 0.3 | 0.1 |
| **T_4_** | **400 (mg/Kg)** | 7.0 | 1.5 | 0.5 | 4.0 | 0.1 | 0.1 |
| **T_5_** | **1% CPB** | 35.7 | 8.5 | 2.7 | 27.0 | 1.0 | 0.4 |
| **T_6_** | **1% CPB + 200**  **(mg/Kg)** | 32.0 | 5.3 | 2.1 | 25.7 | 1.2 | 0.4 |
| **T_7_** | **1% CPB + 300**  **(mg/Kg)** | 18.3 | 4.7 | 1.2 | 11.0 | 1.0 | 0.3 |
| **T_8_** | **1% CPB + 400**  **(mg/Kg)** | 16.3 | 2.3 | 0.9 | 9.3 | 0.3 | 0.1 |
| **T_9_** | **2% CPB**  **(mg/Kg)** | 40.0 | 10.3 | 3.6 | 32.0 | 1.8 | 0.4 |
| **T_10_** | **2% CPB + 200**  **(mg/Kg)** | 33.0 | 7.5 | 3.1 | 36.7 | 1.6 | 0.8 |
| **T_11_** | **2% CPB + 300**  **(mg/Kg)** | 22.0 | 5.5 | 1.6 | 16.3 | 1.1 | 0.4 |
| **T_12_** | **2% CPB + 400**  **(mg/Kg)** | 21 | 3.2 | 1.4 | 16.7 | 0.4 | 0.2 |

Values are mean of the three replicates

**Table S4:** Effect of soil amendment with plant biomass of Cloncurry buffel grass (CPB) on growth attributes of 60-day old tomato plant under Cr(VI).

| **Treatments** | **Concentration** | **Shoot length**  **(cm)** | **Shoot fresh weight**  **(g)** | **Shoot dry**  **weight**  **(g)** | **Root**  **length**  **(cm)** | **Root**  **fresh weight**  **(g)** | **Root**  **dry**  **weight**  **(g)** |
| --- | --- | --- | --- | --- | --- | --- | --- |
| **T_1_** | **0** | 34.0 | 5.9 | 2.0 | 28.0 | 0.9 | 0.3 |
| **T_2_** | **200 (mg/Kg)** | 13.33 | 2.03 | 0.63 | 9.0 | 0.30 | 0.11 |
| **T_3_** | **300 (mg/Kg)** | 7.00 | 1.23 | 0.31 | 5.0 | 0.31 | 0.10 |
| **T_4_** | **400 (mg/Kg)** | 6.00 | 0.98 | 0.20 | 4.3 | 0.09 | 0.03 |
| **T_5_** | **1% CPB** | 35.7 | 8.5 | 2.7 | 27.0 | 1.0 | 0.4 |
| **T_6_** | **1% CPB + 200**  **(mg/Kg)** | 24.3 | 5.60 | 1.60 | 23.0 | 0.72 | 0.22 |
| **T_7_** | **1% CPB + 300**  **(mg/Kg)** | 17.7 | 3.70 | 0.82 | 16.0 | 0.68 | 0.12 |
| **T_8_** | **1% CPB + 400**  **(mg/Kg)** | 9.3 | 1.24 | 0.41 | 17.0 | 0.09 | 0.04 |
| **T_9_** | **2% CPB**  **(mg/Kg)** | 40.0 | 10.3 | 3.6 | 32.0 | 1.8 | 0.4 |
| **T_10_** | **2% CPB + 200**  **(mg/Kg)** | 24.7 | 6.03 | 1.80 | 25.0 | 0.89 | 0.23 |
| **T_11_** | **2% CPB + 300**  **(mg/Kg)** | 16.7 | 4.16 | 0.87 | 21.0 | 0.80 | 0.15 |
| **T_12_** | **2% CPB + 400**  **(mg/Kg)** | 9.7 | 1.65 | 0.57 | 16.3 | 0.11 | 0.04 |

Values are the mean of three replicates.

**Table S5:** Metal uptake by different parts of tomato plant due to soil amendment with plant biomass of Cloncurry buffel grass (CPB) under Cr(III) (a) and Cr(VI) (b) stress.

| **Metal** | **Concentration** | **CPB** | **Root** | **Stem** | **Leaves** |
| --- | --- | --- | --- | --- | --- |
| **Cr(III) mg/Kg** | **200** |  | 80 | 50 | 40 |
|  |  | **1% CPB** | 39 | 20 | 6 |
|  |  | **2% CPB** | 24 | 10 | 3 |
|  | **300** |  | 104 | 80 | 56 |
|  |  | **1% CPB** | 64 | 31 | 17 |
|  |  | **2% CPB** | 47 | 22 | 10 |
|  | **400** |  | 140 | 100 | 70 |
|  |  | **1% CPB** | 90 | 47 | 24 |
|  |  | **2% CPB** | 75 | 36 | 17 |
| **Cr(VI) mg/Kg** | **200** |  | 74 | 56 | 45 |
|  |  | **1% CPB** | 70 | 35 | 10 |
|  |  | **2% CPB** | 45 | 20 | 9 |
|  | **300** |  | 110 | 96 | 55 |
|  |  | **1% CPB** | 90 | 50 | 25 |
|  |  | **2% CPB** | 55 | 31 | 16 |
|  | **400** |  | 140 | 110 | 75 |
|  |  | **1% CPB** | 96 | 60 | 30 |
|  |  | **2% CPB** | 82 | 43 | 31 |

Values are the mean of three replicates.

**Table S6:** Translocation factor and bioconcentration factor of the tomato plant.

| **Metal** | **Concentration** | **CPB** | **Translocation factor** | **Bioconcentration factor** |
| --- | --- | --- | --- | --- |
| **Cr(III) mg/Kg** | **200** |  | 1.12 | 64.86 |
|  |  | **1% CPB** | 0.67 | 14.44 |
|  |  | **2% CPB** | 0.54 | 5.46 |
|  | **300** |  | 1.31 | 163.40 |
|  |  | **1% CPB** | 0.75 | 41.38 |
|  |  | **2% CPB** | 0.68 | 21.49 |
|  | **400** |  | 1.21 | 285.03 |
|  |  | **1% CPB** | 0.79 | 83.53 |
|  |  | **2% CPB** | 0.71 | 44.56 |
| **Cr(VI) mg/Kg** | **200** |  | 1.36 | 111.12 |
|  |  | **1% CPB** | 0.64 | 36.04 |
|  |  | **2% CPB** | 0.64 | 22.21 |
|  | **300** |  | 1.37 | 310.99 |
|  |  | **1% CPB** | 0.83 | 93.36 |
|  |  | **2% CPB** | 0.85 | 53.72 |
|  | **400** |  | 1.32 | 576.84 |
|  |  | **1% CPB** | 0.94 | 212.08 |
|  |  | **2% CPB** | 0.90 | 135.44 |

Values are the mean of three replicates.
